# A Construction of Optimal One-Coincidence Frequency-Hopping Sequences via Generalized Cyclotomy

**DOI:** 10.3390/e26110935

**Published:** 2024-10-31

**Authors:** Minfeng Shao, Ying Miao

**Affiliations:** 1 School of Computer and Software Engineering, Xihua University, Chengdu 610039, China; 2 Graduate School of Systems and Information Engineering, University of Tsukuba, Tennodai 1-1-1, Tsukuba 305-8573, Japan; miao@sk.tsukaba.ac.jp

**Keywords:** frequency-hopping sequence, Hamming correlation, Peng–Fan bound, generalized cyclotomy

## Abstract

Frequency-hopping sequences (FHSs) with low Hamming correlation are essential for synchronization and multiple-access communication systems. In this paper, we propose a novel construction of FHSs using generalized cyclotomy. Our results reveal that the constructed FHSs exhibit a one-coincidence property, meaning that the smallest correlation between different FHSs, aside from the trivial case, is minimized. Additionally, the new sets of FHSs achieve an optimal size in relation to a known theoretical bound.

## 1. Introduction

Frequency-hopping multiple access (FHMA) is widely applied in modern communication systems, including military communications, ultrawideband (UWB), and Bluetooth [1]. In these systems, a set of frequency-hopping patterns is essential for minimizing the maximum Hamming out-of-phase autocorrelation and cross-correlation, which aids in signal differentiation and reduces multiple-access interference. To achieve this, it is desirable to use frequency-hopping sequences (FHSs) that meet the Lempel–Greenberger bound or sets of FHSs that meet the Peng–Fan bound or the coding theory bounds for FHS sets. There exist several combinatorial and algebraic constructions of optimal FHSs and FHS sets (see [2,3,4,5,6,7,8,9,10,11,12,13,14,15,16,17,18,19,20,21], etc.).

The maximum correlation between FHSs is always greater than or equal to one, except in the trivial case where no frequency is repeated across the entire set of FHSs. As extreme cases, a set of FHSs with a maximum correlation of one is known as a one-coincidence FHS set, or simply one-coincidence FHSs. However, compared to optimal FHSs, our understanding of constructions of one-coincidence FHSs is relatively limited. For known constructions for one-coincidence FHSs, the reader may refer to [4,22,23] and the references therein.

In the literature, one of the most significant methods for constructing frequency-hopping sequences (FHSs) is cyclotomy. The classical cyclotomic cosets over $F_{p}$ have been employed to design FHSs with optimal autocorrelation properties [5] and optimal sets of FHSs [7]. In [6], *k*-fold cyclotomic cosets were used to create both optimal FHSs and optimal FHS sets. By dividing an *m*-sequence into cyclotomic cosets, one can generate optimal FHSs and sets of FHSs [10,11]. Additionally, in [24], Zeng et al. introduced a new form of generalized cyclotomy over $Z_{n}$ to construct optimal FHSs and FHS sets. For one-coincidence FHSs, cyclotomic cosets over $F_{q}$ have been utilized to develop optimal constructions [4]. However, it remains an open question whether generalized cyclotomic cosets can always yield one-coincidence FHSs.

This paper aims to explore the application of generalized cyclotomic cosets in constructing one-coincidence FHSs. Specifically, we apply the generalized cyclotomic cosets defined in [24] to build one-coincidence FHSs. Our results demonstrate that these cosets can also produce one-coincidence FHS sets with the maximum number of sequences with respect to a known theoretic bound [4].

The rest of this paper is structured as follows: In Section 2, we provide some preliminaries necessary for the discussion. Section 3 introduces a construction of one-coincidence FHSs via generalized cyclotomic cosets. Section 4 concludes the paper with some remarks.

## 2. Preliminaries

In this section, we introduce some necessary preliminaries on frequency-hopping sequences. Let *l* be a positive integer and $F=\{f_{0},f_{1},\ldots ,f_{l-1}\}$ represent a set of *l* available frequencies, also referred to as the *alphabet*. A sequence $X={\left\{x_{t}\right\}}_{t=0}^{n-1}$ is called a *frequency-hopping sequence* (FHS) of length *n* over F if $x_{t}\in F$ for all 0≤t≤n−1.

For two FHSs $X={\left\{x_{t}\right\}}_{t=0}^{n-1}$ and $Y={\left\{y_{t}\right\}}_{t=0}^{n-1}$, both of length *n* over F, their *Hamming correlation* $H_{X,Y}$ is defined as follows:

$$H_{X,Y}\left(\tau \right)=\sum_{t=0}^{n-1}h\left[x_{t},y_{t+\tau }\right],\;0\leq \tau \leq n-1,$$

where h[a,b]=1 if a=b, and 0 otherwise. All operations on the position indices are performed modulo *n*.

If there exists an integer *k* such that $x_{t}=y_{t+k}$ for all 0≤t≤n−1, then *X* and *Y* are said to be *cyclic shift equivalent*, with all position index operations modulo *n*. If k≡0(modn), then we say X=Y, and in this case, $H_{X,Y}\left(\tau \right)$ is called the *Hamming autocorrelation* of *X*, denoted by $H_{X}\left(\tau \right)$.

Define H(X) for an FHS *X* as follows:

$$H\left(X\right)=\max_{1\leq \tau \leq n-1}\left\{H_{X}\left(\tau \right)\right\},$$

and for two distinct FHSs *X* and *Y* (i.e., X≠Y), define H(X,Y) as follows:

$$H\left(X,Y\right)=\max_{0\leq \tau \leq n-1}\left\{H_{X,Y}\left(\tau \right)\right\}.$$

For a∈R, let ⌈a⌉ denote the smallest integer greater than or equal to *a*. A lower bound of H(X) was established by Lempel and Greenberger as below.

**Lemma** **1** ([18]). *Let X be a frequency-hopping sequence of length n over an alphabet with size l. Then, we have*

$$H\left(X\right)\geq \left\lceil \frac{\left(n-\epsilon )(n+\epsilon -l\right)}{l(n-1)}\right\rceil ,$$

*where ϵ is the least non-negative residue of n modulo l.*

An (n,l,λ) FHS *X* is called optimal if it meets the Lempel–Greenberger bound in Lemma 1 with equality.

Let S be a set of *N* frequency-hopping sequences with length *n* over an alphabet F with |F|=l. The *maximum nontrivial Hamming correlation*M(S) of the sequence set S is defined as

$$M\left(S\right)=\max\left\{\max_{X\in S}H\left(X\right),\max_{X,Y\in S,X\neq Y}H\left(X,Y\right)\right\}.$$

In this case, we say that the set S has parameters (n,N,λ;l) (denoted as (n,N,λ;l) FHS set for short), where λ=M(S).

**Lemma** **2** ([25]). *Let S be an (n,N,λ;l) FHS set. Define I=⌊nN/l⌋. Then*

(1) $$M\left(S\right)\geq \left\lceil \frac{(nN-l)n}{(nN-1)l}\right\rceil$$

*and*

(2) $$M\left(S\right)\geq \left\lceil \frac{2InN-(I+1)Il}{(nN-1)N}\right\rceil .$$

In this paper, an (n,N,λ;l) FHS set is called *optimal* if one of the Peng–Fan bounds in Lemma 2 is met. An FHS set S is said to be one-coincidence if M(S)=1.

**Remark** **1.** *According to Lemma 2, when nN−l>0 we have M(S)≥1. This is to say that all one-coincidence FHS sets are optimal provided that nN−l>0.*

In [4], a theoretic bound on the size of one-coincidence FHS sets is introduced as follows.

**Lemma** **3** ([4]). *Let S be an (n,N,1;l) one-coincidence FHS set. Then, we have*

$$N\leq \frac{l(l-1)}{n}.$$

**Definition** **1.** *A one-coincidence FHS set is said to have an optimal size if its size N meets the upper bound in Lemma 3 exactly.*

## 3. A New Construction of One-Coincidence FHSs

In [7,8,24], generalized cyclotomy was included to construct sets of FHSs. In this section, Zeng–Cai–Tang–Yang cyclotomy [24] is applied to construct sets of FHSs with one-coincidence property.

Firstly, we recall the definition of Zeng–Cai-Tang–Yang generalized cyclotomy. For an odd positive integer *v*, let $v=p_{1}^{m_{1}}p_{2}^{m_{2}}\cdots p_{t}^{m_{t}}$, where $p_{1},p_{2},\ldots ,p_{t}$ are distinct primes with $2<p_{1}<p_{2}<\cdots <p_{t}$ and $m_{1},m_{2},\ldots ,m_{t}$ are positive integers. Let $Z_{v}^{*}$ denote the set of invertible elements in $Z_{v}$, i.e., $Z_{v}^{*}=\left\{x\in Z_{v}|\gcd\left(x,v\right)=1\right\}.$ For any subset *D* of $Z_{v}$ and any element $a\in Z_{v}$, define aD≜{ah∣h∈D}, where the multiplication is performed in $Z_{v}$.

A *partition* $\left\{D_{0},D_{1},\ldots ,D_{d-1}\right\}$ of $Z_{v}^{*}$ is a collection of subsets of $Z_{v}^{*}$ such that

$$D_{i}\cap D_{j}=\emptyset \;\;\mathrm{for}\;\mathrm{any}\;\;i\neq j,\;\;\mathrm{and}\;\;\overset{d-1}{\underset{i=0}{⋃}}D_{i}=Z_{v}^{*}.$$

If $D_{0}$ is a multiplicative subgroup of $Z_{v}^{*}$ and $D_{i}=a_{i}D_{0}$ for some elements $a_{i}\in Z_{v}^{*}$, where 1≤i≤d−1, then the sets $D_{0},D_{1},\ldots ,D_{d-1}$ are called the *generalized cyclotomic classes* of order *d* when *v* is composite, and *classical cyclotomic classes* of order *d*, when *v* is prime [9].

Let φ(v) be the well-known Euler function, which counts the number of positive integers less than *v* that are coprime to *v*. An element $\beta \in Z_{v}^{*}$ is called a *primitive root modulov* if its multiplicative order modulo *v* equals to φ(v). Recall that if a primitive root modulo *v* exists, then we have $v\in \{2,4,p^{t},2p^{t}$, where *t* is a positive integer and *p* is an odd prime [26]. It is well known that when *p* is an odd prime there exists at least one element $\delta \in Z_{p}$, which is a primitive root modulo $p^{t}$ for all t≥1 [26].

For each 1≤i≤t, let $\delta_{i}$ be a primitive root modulo $p_{i}^{j}$ for all j≥1. By the Chinese Remainder Theorem [26], for each 1≤i≤t, there exists a unique $\beta_{i}\in Z_{v}^{*}$ such that

$$\beta_{i}\equiv \left\{\begin{array}{c} \delta_{i}\;\left(\mathrm{mod}\;p_{i}^{m_{i}}\right),\\ 1\;(\mathrm{mod}\;p_{j}^{m_{j}})\;\;\mathrm{for}\;\;j\neq i\;\;\mathrm{and}\;\;1\leq j\leq t. \end{array}\right.$$

Let e>1 be a factor of $\gcd(p_{1}-1,p_{2}-1,\ldots ,p_{t}-1)$, i.e., $p_{i}-1=ef_{i},\;\;\;e>1,$ for some positive integers $f_{i}$, where 1≤i≤t. Consider a factor $v_{1}=p_{i_{1}}^{m_{i_{1}}^{\prime }}p_{i_{2}}^{m_{i_{2}}^{\prime }}\cdots p_{i_{t(v_{1})}}^{m_{i_{t(v_{1})}}^{\prime }}$ of *v*, where $t(v_{1})$ denotes the number of prime factors of $v_{1}$ and $\left\{p_{i_{j}}|1\leq j\leq t\left(v_{1}\right)\right\}\subseteq \left\{p_{i}|1\leq i\leq t\right\}$ with $0<m_{i_{j}}^{\prime }\leq m_{i_{j}}$. Again, by the Chinese Remainder Theorem, there exists a unique $\beta_{\left(v_{1}\right)}\in Z_{v_{1}}^{*}$ such that

$$\beta_{\left(v_{1}\right)}\equiv \delta_{i_{j}}^{f_{i_{j}}p_{i_{j}}^{m_{i_{j}}^{\prime }-1}}\;\left(\mathrm{mod}\;p_{i_{j}}^{m_{i_{j}}^{\prime }}\right),\;\;\mathrm{for}\;\mathrm{all}\;\;1\leq j\leq t\left(v_{1}\right).$$

It is straightforward to verify that the multiplicative order of $\beta_{\left(v_{1}\right)}$ modulo $v_{1}$ is *e*, which implies that the set

$$D^{\left(v_{1}\right)}=\left\{\beta_{\left(v_{1}\right)}^{t}|0\leq t\leq e-1\right\}$$

is a cyclic subgroup of $Z_{v_{1}}^{*}$ of order *e*.

For any

$$I_{v_{1}}=\left(\tau_{1},\tau_{2},\ldots ,\tau_{t(v_{1})}\right)\in I\left(v_{1}\right)≜\left\{0\leq \tau_{1}\leq f_{i_{1}}p_{i_{1}}^{m_{i_{1}}^{\prime }-1}-1\right\}\times Z_{\varphi (p_{i_{2}}^{m_{i_{2}}^{\prime }})}\times \cdots \times Z_{\varphi (p_{t(v_{1})}^{m_{t(v_{1})}^{\prime }})},$$

define $D_{I_{v_{1}}}^{\left(v_{1}\right)}$ as

$$D_{I_{v_{1}}}^{\left(v_{1}\right)}=\Delta_{\left(v_{1}\right)}^{I_{v_{1}}}D^{\left(v_{1}\right)}≜\delta_{i_{1}}^{\tau_{1}}\delta_{i_{2}}^{\tau_{2}}\cdots \delta_{i_{t(v_{1})}}^{\tau_{t(v_{1})}}D^{\left(v_{1}\right)}.$$

As shown in [24], the sets $D_{I_{v_{1}}}^{\left(v_{1}\right)}$ for $I_{v_{1}}\in I\left(v_{1}\right)$ are the generalized cyclotomic classes of order $\frac{\varphi (v_{1})}{e}$ with respect to $v_{1}$. Finally, it can be checked that

$$\Psi ≜\underset{1<v_{1},v_{1}|v}{⋃}\underset{I_{v_{1}}\in I\left(v_{1}\right)}{⋃}\frac{v}{v_{1}}D_{I_{v_{1}}}^{\left(v_{1}\right)}$$

is a partition of $Z_{v}\setminus \left\{0\right\}$, consisting of $\frac{v-1}{e}$ sets.

**Construction** **1.** *Let* η∈Ψ *, where* $\eta =\frac{v}{v_{1}}\Delta_{\left(v_{1}\right)}^{I_{v_{1}}}$ *with* $1<v_{1},v_{1}|v$ *and* $I_{v_{1}}\in I\left(v_{1}\right)$ *. Define a sequence* $S_{\eta }$ *with length e as*

$$S_{\eta }≜\left(\eta \beta_{\left(v_{1}\right)}^{0},\eta \beta_{\left(v_{1}\right)}^{1},\cdots ,\eta \beta_{\left(v_{1}\right)}^{e-1}\right).$$

*For any* $a\in Z_{v}$ *, define*

(3) $$\begin{array}{cc} S_{\eta ,a}= & S_{\eta }+a\\ ≜ & \left(\eta \beta_{\left(v_{1}\right)}^{0}+a,\eta \beta_{\left(v_{1}\right)}^{1}+a,\cdots ,\eta \beta_{\left(v_{1}\right)}^{e-1}+a\right). \end{array}$$

*Let* $S=\{S_{\eta ,a}\;|\;\eta \in \Psi ,a\in Z_{v}\}$ *be the set of* $\frac{v(v-1)}{e}$ *frequency-hopping sequences over* $Z_{v}$.

**Theorem** **1.** *Let $v=p_{1}^{m_{1}}p_{2}^{m_{2}}\cdots p_{t}^{m_{t}}$ and $e|(p_{i}-1)$ for all 1≤i≤t, where $p_{1}$, $p_{2}$, …, and $p_{t}$ are t primes with $2<p_{1}<p_{2}<\cdots <p_{t}$, and $m_{1}$, $m_{2}$, ⋯, and $m_{t}$ are positive integers. The FHS set S generated by Construction 1 is an optimal one-coincidence FHS set with parameters $\left(e,\frac{v(v-1)}{e},1;v\right)$. Furthermore, the one-coincidence FHS set has an optimal size with respect to the bound in Lemma 3.*

**Proof.**  By Construction 1, to prove S is an optimal one-coincidence FHS set with parameters $\left(e,\frac{v(v-1)}{e},1;v\right)$, it suffices to show that M(S)≤1, i.e., for any $a_{1},a_{2}\in Z_{v}$ and $\beta_{1},\beta_{2}\in \Psi$ with $\left(a_{1},\beta_{1}\right)\neq \left(a_{2},\beta_{2}\right)$, we have

(4) $$H_{S_{\eta_{1},a_{1}}}\left(\tau \right)\leq 1\;\mathrm{for}\;\;1\leq \tau \leq e-1,$$

and

(5) $$H_{S_{\eta_{1},a_{1}},S_{\eta_{2},a_{2}}}\left(\tau \right)\leq 1\;\mathrm{for}\;\;0\leq \tau \leq e-1.$$

Note that by (3), we have

$$H_{S_{\eta ,a}}\left(\tau \right)=\sum_{0\leq j\leq e-1}h\left[\eta \beta_{\left(v_{1}\right)}^{j},\eta \beta_{\left(v_{1}\right)}^{j+\tau }\right]=0$$

for any 1≤τ≤e−1, since $\beta_{\left(v_{1}\right)}$ has order *e* for any $v_{1}|v$ and $v_{1}>1$. This implies that (4) holds for any $S_{\eta ,a}\in S$.

To address the inequality in (5), assume, for contradiction, that $H_{S_{\eta_{1},a_{1}},S_{\eta_{2},a_{2}}}\geq 2$ for some $a_{1},a_{2}\in Z_{v}$, i.e.,

$$H_{S_{\eta_{1},a_{1}},S_{\eta_{2},a_{2}}}\left(\tau \right)=\sum_{0\leq j\leq e-1}h\left[\eta_{1}\beta_{\left(v_{1}\right)}^{j}+a_{1},\eta_{2}\beta_{\left(v_{2}\right)}^{j+\tau }+a_{2}\right]\geq 2.$$

Thus, there exist three integers $0\leq j_{1},$$j_{2},$τ≤e−1 with $j_{1}\neq j_{2}$ such that

$$\eta_{2}\beta_{\left(v_{2}\right)}^{j_{1}+\tau }+a_{2}=\eta_{1}\beta_{\left(v_{1}\right)}^{j_{1}}+a_{1}$$

and

$$\eta_{2}\beta_{\left(v_{2}\right)}^{j_{2}+\tau }+a_{2}=\eta_{1}\beta_{\left(v_{1}\right)}^{j_{2}}+a_{1}.$$

The above two equations imply that

(6) $$\eta_{2}\beta_{\left(v_{2}\right)}^{j_{1}+\tau }-\eta_{2}\beta_{\left(v_{2}\right)}^{j_{2}+\tau }=\eta_{1}\beta_{\left(v_{1}\right)}^{j_{1}}-\eta_{1}\beta_{\left(v_{1}\right)}^{j_{2}}.$$

We now distinguish two cases to demonstrate that (6) leads to a contradiction.

Case I: $\left(\eta_{1},\eta_{2}\right)=\left(\frac{v}{v_{1}}\Delta_{\left(v_{1}\right)}^{I_{v_{1}}},\frac{v}{v_{2}}\Delta_{\left(v_{2}\right)}^{I_{v_{2}}}\right)$ with $v_{1}\neq v_{2}$. Since $v_{1}|v$, $v_{2}|v$, and $v_{1}\neq v_{2}$, there must exist at least one index 1≤i≤t such that $0\leq m_{i}^{\prime }\neq m_{i}^{*}\leq m_{i}$, where $v_{1}=p_{1}^{m_{1}^{\prime }}p_{2}^{m_{2}^{\prime }}\cdots p_{t}^{m_{t}^{\prime }}$ and $v_{2}=p_{1}^{m_{1}^{*}}p_{2}^{m_{2}^{*}}\cdots p_{t}^{m_{t}^{*}}$. Without loss of generality, assume $m_{i}^{\prime }>m_{i}^{*}$. By (6), we have

$$\begin{array}{cc} \; & \frac{v}{v_{2}}\Delta_{\left(v_{2}\right)}^{I_{v_{2}}}\beta_{\left(v_{2}\right)}^{j_{1}+\tau }-\frac{v}{v_{2}}\Delta_{\left(v_{2}\right)}^{I_{v_{2}}}\beta_{\left(v_{2}\right)}^{j_{2}+\tau }\\ \equiv & \frac{v}{v_{1}}\Delta_{\left(v_{1}\right)}^{I_{v_{1}}}\beta_{\left(v_{1}\right)}^{j_{1}}-\frac{v}{v_{1}}\Delta_{\left(v_{1}\right)}^{I_{v_{1}}}\beta_{\left(v_{1}\right)}^{j_{2}}\;\left(\mathrm{mod}\;p_{i}^{m_{i}}\right), \end{array}$$

which means

$$\begin{array}{cc} \; & \prod_{1\leq j\leq t}p_{j}^{m_{j}-m_{j}^{*}}\Delta_{\left(v_{2}\right)}^{I_{v_{2}}}\left(\beta_{\left(v_{2}\right)}^{j_{1}+\tau }-\beta_{\left(v_{2}\right)}^{j_{2}+\tau }\right)\\ \equiv & \prod_{1\leq j\leq t}p_{j}^{m_{j}-m_{j}^{\prime }}\Delta_{\left(v_{1}\right)}^{I_{v_{1}}}\left(\beta_{\left(v_{1}\right)}^{j_{1}}-\beta_{\left(v_{1}\right)}^{j_{2}}\right)\;\left(\mathrm{mod}\;p_{i}^{m_{i}}\right). \end{array}$$

The preceding congruence hints that

(7) $$\begin{array}{cc} \; & p_{i}^{m_{i}^{\prime }-m_{i}^{*}}\prod_{\begin{array}{c} 1\leq j\leq t\\ j\neq i \end{array}}p_{j}^{m_{j}-m_{j}^{*}}\Delta_{\left(v_{2}\right)}^{I_{v_{2}}}\left(\beta_{\left(v_{2}\right)}^{j_{1}+\tau }-\beta_{\left(v_{2}\right)}^{j_{2}+\tau }\right)\\ \equiv & \prod_{\begin{array}{c} 1\leq j\leq t\\ j\neq i \end{array}}p_{j}^{m_{j}-m_{j}^{\prime }}\Delta_{\left(v_{1}\right)}^{I_{v_{1}}}\left(\beta_{\left(v_{1}\right)}^{j_{1}}-\beta_{\left(v_{1}\right)}^{j_{2}}\right)\;\left(\mathrm{mod}\;p_{i}^{m_{i}^{\prime }}\right). \end{array}$$

Using (7) and the fact that $\gcd(p_{i},p_{j})=1$ for 1≤i,j≤t with i≠j, we conclude that

$$0\equiv \beta_{\left(v_{1}\right)}^{j_{1}}-\beta_{\left(v_{1}\right)}^{j_{2}}\;\left(\mathrm{mod}\;p_{i}\right),$$

which implies

$$\delta_{i}^{j_{1}f_{i}}-\delta_{i}^{j_{2}f_{i}}\equiv 0\;\left(\mathrm{mod}\;p_{i}\right),$$

contradicting the fact that $\delta_{i}$ has order $ef_{i}$ and $0\leq j_{1},j_{2}\leq e-1$ with $j_{1}\neq j_{2}$.

Case II: $\left(\eta_{1},\eta_{2}\right)=\left(\frac{v}{v_{1}}\Delta_{\left(v_{1}\right)}^{I_{v_{1}}},\frac{v}{v_{1}}\Delta_{\left(v_{1}\right)}^{I_{v_{1}}^{\prime }}\right)$. In this case, (6) can be rewritten as

$$\begin{array}{cc} \; & \frac{v}{v_{1}}\Delta_{\left(v_{1}\right)}^{I_{v_{1}}^{\prime }}\left(\beta_{\left(v_{1}\right)}^{j_{1}+\tau }-\beta_{\left(v_{1}\right)}^{j_{2}+\tau }\right)\\ \equiv & \frac{v}{v_{1}}\Delta_{\left(v_{1}\right)}^{I_{v_{1}}}\left(\beta_{\left(v_{1}\right)}^{j_{1}}-\beta_{\left(v_{1}\right)}^{j_{2}}\right)\;\left(\mathrm{mod}\;v\right), \end{array}$$

which means

(8) $$\Delta_{\left(v_{1}\right)}^{I_{v_{1}}^{\prime }}\left(\beta_{\left(v_{1}\right)}^{j_{1}+\tau }-\beta_{\left(v_{1}\right)}^{j_{2}+\tau }\right)\equiv \Delta_{\left(v_{1}\right)}^{I_{v_{1}}}\left(\beta_{\left(v_{1}\right)}^{j_{1}}-\beta_{\left(v_{1}\right)}^{j_{2}}\right)\;\left(\mathrm{mod}\;v_{1}\right).$$

Let

$$I_{v_{1}}=\left(\tau_{0},\tau_{1},\cdots ,\tau_{t(v_{1})}\right)\neq I_{v_{1}}^{\prime }=\left(\tau_{0}^{\prime },\tau_{1}^{\prime },\cdots ,\tau_{t(v_{1})}^{\prime }\right)$$

and $v_{1}=p_{i_{0}}^{m_{i_{0}}}p_{i_{1}}^{m_{i_{1}}},\cdots ,p_{i_{t(v_{1})-1}}^{m_{i_{t(v_{1})-1}}}.$ Now, recalling (8) we obtain that

$$\begin{array}{cc} \; & \delta_{i_{0}}^{\tau_{0}f_{i_{0}}}\left(\delta_{i_{0}}^{\left(j_{1}+\tau \right)f_{i_{0}}}-\delta_{i_{0}}^{\left(j_{2}+\tau \right)f_{i_{0}}}\right)\\ \equiv & \delta_{i_{0}}^{\tau_{0}^{\prime }f_{i_{0}}}\left(\delta_{i_{0}}^{j_{1}f_{i_{0}}}-\delta_{i_{0}}^{j_{2}f_{i_{0}}}\right)\;\left(\mathrm{mod}\;p_{i_{0}}\right), \end{array}$$

i.e.,

$$\left\{\begin{array}{cc} \delta_{i_{0}}^{\tau_{0}^{\prime }-\tau_{i_{0}}+\tau f_{i_{0}}}\left(\delta_{i_{0}}^{j_{1}f_{i_{0}}}-\delta_{i_{0}}^{j_{2}f_{i_{0}}}\right)\equiv 0\;\left(\mathrm{mod}\;p_{i_{0}}\right) & \;\mathrm{if}\;\tau_{0}\neq \tau_{0}^{\prime }\\ \delta_{i_{0}}^{\tau_{i_{0}}+\tau f_{i_{0}}}\left(\delta_{i_{0}}^{j_{1}f_{i_{0}}}-\delta_{i_{0}}^{j_{2}f_{i_{0}}}\right)\equiv 0\;\left(\mathrm{mod}\;p_{i_{0}}\right)\; & \;\mathrm{otherwise}. \end{array}\right.$$

The preceding congruence enhances that

(9) $$\begin{array}{cc} \; & p_{i}^{m_{i}^{\prime }-m_{i}^{*}}\prod_{1\leq j\leq t,j\neq i}p_{j}^{m_{j}-m_{j}^{*}}\Delta_{\left(v_{2}\right)}^{I_{v_{2}}}\left(\beta_{\left(v_{2}\right)}^{j_{1}+\tau }-\beta_{\left(v_{2}\right)}^{j_{2}+\tau }\right)\\ \equiv & \prod_{1\leq j\leq t,j\neq i}p_{j}^{m_{j}-m_{j}^{\prime }}\delta^{I_{v_{1}}}\left(\beta_{\left(v_{1}\right)}^{j_{1}}-\beta_{\left(v_{1}\right)}^{j_{2}}\right)\;\left(\mathrm{mod}\;p_{i}^{m_{i}^{\prime }}\right) \end{array}$$

By (9) and the fact $\gcd(p_{i},p_{j})=1$ for 1≤i,j≤t and i≠j, we conclude that

$$0\equiv \beta_{\left(v_{1}\right)}^{j_{1}}-\beta_{\left(v_{1}\right)}^{j_{2}}\;\left(\mathrm{mod}\;p_{i}\right),$$

i.e.,

$$\delta_{i}^{j_{1}f_{i}}-\delta_{i}^{j_{2}f_{i}}\equiv 0\;\left(\mathrm{mod}\;p_{i}\right),$$

which contradicts the fact that $\delta_{i}$ has order *e* and $0\leq j_{1},j_{2}\leq e-1$ with $j_{1}\neq j_{2},$ which contradict the fact that $\delta_{i_{0}}$ has multiple order $ef_{i_{0}}$ in $Z_{p_{i_{0}}}$ and $0\leq j_{1},j_{2}\leq e-1$, and $j_{1}\neq j_{2}$.

Therefore, combining the above two cases, we have

$$H_{S_{\eta_{1},a_{1}},S_{\eta_{2},a_{2}}}\left(\tau \right)\leq 1$$

for any $S_{\eta_{1},a_{1}},S_{\eta_{2},a_{2}}\in S$ and $S_{\eta_{1},a_{1}}\neq S_{\eta_{2},a_{2}}.$ Thus, we know that S has parameters $\left(e,\frac{v(v-1)}{e},1;v\right)$, which is optimal according to Remark 1.

Note that $N=\frac{v(v-1)}{e}$, which is exactly the optimal size with respect to the bound in Lemma 3. This completes the proof. □

**Remark** **2.** *In [4], one-coincidence FHS sets with an optimal size were also constructed using cyclotomic cosets over $F_{q}$. These FHS sets have parameters $\left(e,\frac{q(q-1)}{e},1;q\right)$, where q is a prime power. In comparison to this known family of one-coincidence FHS sets with an optimal size, our construction offers more flexibility in terms of parameters, as the alphabet size in our approach can be any odd positive integer.*

**Remark** **3.** *In [24], Zeng–Cai–Tang–Yang cyclotomy was used to construct optimal FHSs and FHS sets. However, for the case of FHS sets, the sequences have a maximum Hamming correlation of at least 2, meaning the constructions in [24] cannot produce one-coincidence FHS sets. In Construction 1, we apply Zeng–Cai–Tang–Yang cyclotomy differently to construct one-coincidence FHS sets with an optimal family size.*

**Remark** **4.** *Since the generalized cyclotomic cosets have a different algebraic structure compared to the cyclotomic cosets over $F_{q}$, our construction does not encompass the one from [4]. For example, let $q=p^{m}$. Then, the known construction generates FHS sets with parameters $\left(e,\frac{q(q-1)}{e},1;q\right)$, where e∣(q−1). In contrast, Construction 1 produces FHS sets with parameters $\left(e,\frac{q(q-1)}{e},1;q\right)$, where e∣(p−1) and $q=p^{m}$.*

## 4. Conclusions

In this paper, a novel construction of FHSs was proposed using generalized cyclotomy. Additionally, the new sets of FHSs achieved an optimal size with respect to a known theoretical bound.

Other types of cyclotomic cosets, such as those introduced in [8,9], have also been explored in the literature. However, applying these generalized cyclotomic cosets to construct one-coincidence FHS sets remains an open question, which we leave for future research. 

## Data Availability

The original contributions presented in the study are included in the article, further inquiries can be directed to the corresponding author.
